# Estimating the severity of landscape degradation in future management scenarios based on modeling the dynamics of Hoor Al-Azim International Wetland in Iran-Iraq border

**DOI:** 10.1038/s41598-024-62649-0

**Published:** 2024-05-24

**Authors:** Sharif Joorabian Shooshtari, Fatemeh Jahanishakib

**Affiliations:** 1 Department of Nature Engineering, Agricultural Sciences and Natural Resources University of Khuzestan, Mollasani, Iran; 2 Environmental Science Department, Faculty of Natural Resources and Environmental Studies, University of Birjand, Birjand, South Khorasan Province Iran

**Keywords:** Temporal-spatial changes, Landscape metrics, Effective variables, Validation, Artificial neural network, Hexagonal units, Ecological modelling, Environmental impact

## Abstract

Temporal and spatial changes in land cover in wetland ecosystems indicate the severity of degradation. Understanding such processes in the past, present, and future might be necessary for managing any type of development plan. Therefore, this research has monitored and analyzed the Hoor Al-Azim International Wetland to determine the orientation of its changes in various future scenarios. Wetland status modeling was conducted using developed hybrid approaches and cellular automata along with evaluating the accuracy of the modeled maps. The dynamics of the landscape were simulated using a higher accuracy approach in three scenarios—Water Conservation, Water Decreasing, and Business-as-Usual- to get the level of degradation of the wetland. The results showed that the amount of water in the wetland has decreased in all three periods, and the salt lands and vegetation have undergone drastic changes. The water bodies experienced a reduction of 148,139 ha between 1985 and 2000, followed by a decrease of 9107 ha during the 2000–2015 period. However, based on the results, these developments are expressed better by the developed hybrid approach than the CA-MC approach and are more reliable for future simulation. The figure of merit index, which assesses the hybrid model's accuracy, yielded a value of 18.12%, while the CA-MC model's accuracy was estimated at 14.42%. The assessment of degradation in hexagonal units showed the least degradation in the water conservation scenario compared with the other two scenarios in 2030.

## Introduction

The dynamics of the landscape refer to the changes and transformations in land use and land cover that have occurred over time and space. These events have continued to occur in recent decades with significant intensity, especially in developing countries^1^. Wetlands stand out as distinctive ecosystems, rich in biodiversity, and are particularly vulnerable to the impacts of land-use changes^2^. Globally, wetlands are ecosystems at high risk of endangerment due to a myriad of complex factors, including land cover changes^3^, farmers’ unsustainable groundwater management practices^4^, water and upstream management, as well as external pressures such as climate change and population growth^5^. Land dynamics modeling is crucial for understanding the historical, current, and future state of landscapes to mitigate threats, assess human-induced damages, and manage land use effectively. These models are valuable for detecting changes related to land cover, including cultivation, land degradation, desertification, coastal changes, urban landscape patterns, deforestation, mining activities, and habitat loss. Land degradation is inherently related to the concept of ecological deterioration or loss of the land's capacity to produce goods and services^6,7^. Wetland degradation is characterized by the loss of wetland areas or disruptions in wetland functionality due to human-induced transformations from wetlands to non-wetland territories^8^. Estimating wetland degradation is a problem in wetland research, and establishing a robust and universal method for evaluating the ecological degradation of the wetland landscape has always been a critical issue^6^. Land degradation assessment methods have evolved from field surveys to newer ecological approaches. Accurate information about land changes in large areas is crucial for sustainable development programs. Analyzing and mapping land cover changes over time helps address economic, social, and environmental problems effectively^9^.

The complexity of modeling land cover changes on a global and regional scale stems from the diverse range of variables and the constraints encountered at the local level^10^. Researchers worldwide have used the capabilities of Land Change Modeler (LCM) tool^11,12–14^. Generally, after ensuring the validity of the model, the models are used for future simulation^15,16^. Several studies^15,17–24^ have obtained predictive maps that illustrate potential future land cover changes under various land management scenarios. The efficiency of models in generating such maps is a crucial topic in earth science research, leading to comparisons between different methods. In Wuhan, a hybrid Logistic-MCE-CA–Markov model was used to assess and predict land use and land cover changes, incorporating spatial variables^25^. In Uttar Pradesh, India, simulation models (MLP_Markov, CA_Markov, ST_Markov) were used to study landscape changes. MLP_Markov showed the best performance, followed by CA_Markov and ST_Markov^26^. In a study on Qeshm Island, three hybrid models were used to simulate land use change. The efficiency of the results was measured with the Figure Of Merit index (FOM). The study found a reduction in mangrove ecosystems due to ongoing construction, as indicated by the superior hybrid model^27^.

Although the modeling of land dynamics is the basis of many research studies on landscape planning, the important challenge is how to validate the output of these models^28^ and utilizing them to create a correct understanding in managers and decision makers of the landscape degradation process. Few studies have undertaken a comparative analysis of the precision of hybrid and CA-MC models in forecasting future landscape scenarios and assessing landscape degradation, employing landscape metrics in hexagonal units. Several studies have evaluated degradation based on a statistical analysis at 1672 field sites. Several effective indicators have been identified for various land degradation processes, including rain, slope, plant cover, land abandonment rate, land use intensity, and policy implementation level^29^. Shen et al.^30^ constructed a composite wetland degradation index comprising five indices: wetland surface change rate, landscape fractal dimension, landscape fragmentation, and plant biomass and vegetation coverage. Also, Das and Basu^31^ assessed the wetland ecological degradation through importance-performance analysis (IPA) and landscape metrics. Land use changes detection and degradation using the LCM model and landscape metrics in Bakhtegan, Tashk, and Maharloo lakes showed that the conversion of rangelands and forests to agriculture and construction and the decreasing trend of the water body of the lakes caused degradation of the lake and an increase in salted lands^32^. Peng et al.^33^ used random forest (RF), CLUE-S, and multi-objective programming (MOP) models to simulate four optimal scenarios for 2035 to protect wetlands and SDGs. Indicators of land degradation included land cover, land productivity and carbon stocks^33^.

Research has used landscape metrics to assess degradation, but there is a lack of a systematic methodology to quantify the severity of degradation in specific units. A gap in research exists regarding the utilization of validated modeling outputs to determine the severity of degradation in hexagonal units of wetland ecosystems. This article focuses on evaluating the future degradation of the Hoor Al-Azim wetland, an important transboundary wetland between Iran and Iraq. Through the modeling of land's temporal and spatial dynamics and validating the results, the research aims to provide early warnings to stakeholders and prevent irreversible events. Monitoring and analyzing changes in the wetland over time are crucial for developing management strategies, preserving ecosystem services, such as dust particle prevention and habitat for migratory birds, and promoting job creation and agricultural growth^34^. The consequences of drying up the wetland and turning it from a dust-absorbing area into a dust-producing source have been anthropogenic and destructive for the lives of humanity, plants, and animals. Therefore, the changes in land cover and the way of managing land leads to numerous environmental consequences that require investigation to determine how they will impact the future of the land. One motivation for choosing Hoor Al-Azim wetland for modeling the changes is to reveal the time and place of these changes in the land and to help the wise decision-making process of the managers. Therefore, it is necessary to monitor and simulate the dynamics of wetland changes from the past to the future under various scenarios. It is hoped that it will contribute to the national and international efforts to protect and restore the wetland. Despite modeling the temporal-spatial dynamics of the land by using the developed and proposed method, this article seeks to validate the results of the models. To the best of our knowledge, this is the first study to compare land cover change simulations between a hybrid approach and the CA-MC model in this international wetland. We investigated the dynamics of these changes to evaluate the intensity of degradation using landscape metrics, rendering our process both unique and practical. Therefore, the main research questions of this study were: (1) Which driver variable has the highest impact on the changes in the wetland? (2) What will be the future spatial patterns of land cover change towards the year 2030 under various scenarios? (3) What is the landscape degradation assessment in future scenarios? Also, the goals of this research are:

1. Monitoring changes in the land cover of Horalazim wetland using satellite images,
2. Determining the most influential variable in expressing wetland land cover changes using Cramer's coefficient and producing transition potential maps using artificial neural network,
3. Modeling the status of the wetland with developed hybrid approaches (combination of transition potential maps with the artificial neural network, Markov chain analysis, and multi-objective optimization) and CA-MC,
4. Assessing the accuracy of the modeled maps and simulating the future scenarios of the studied area with a superior approach,
5. Evaluating land degradation in future scenarios.

## Materials and methods

### Study area

**Figure 1:**
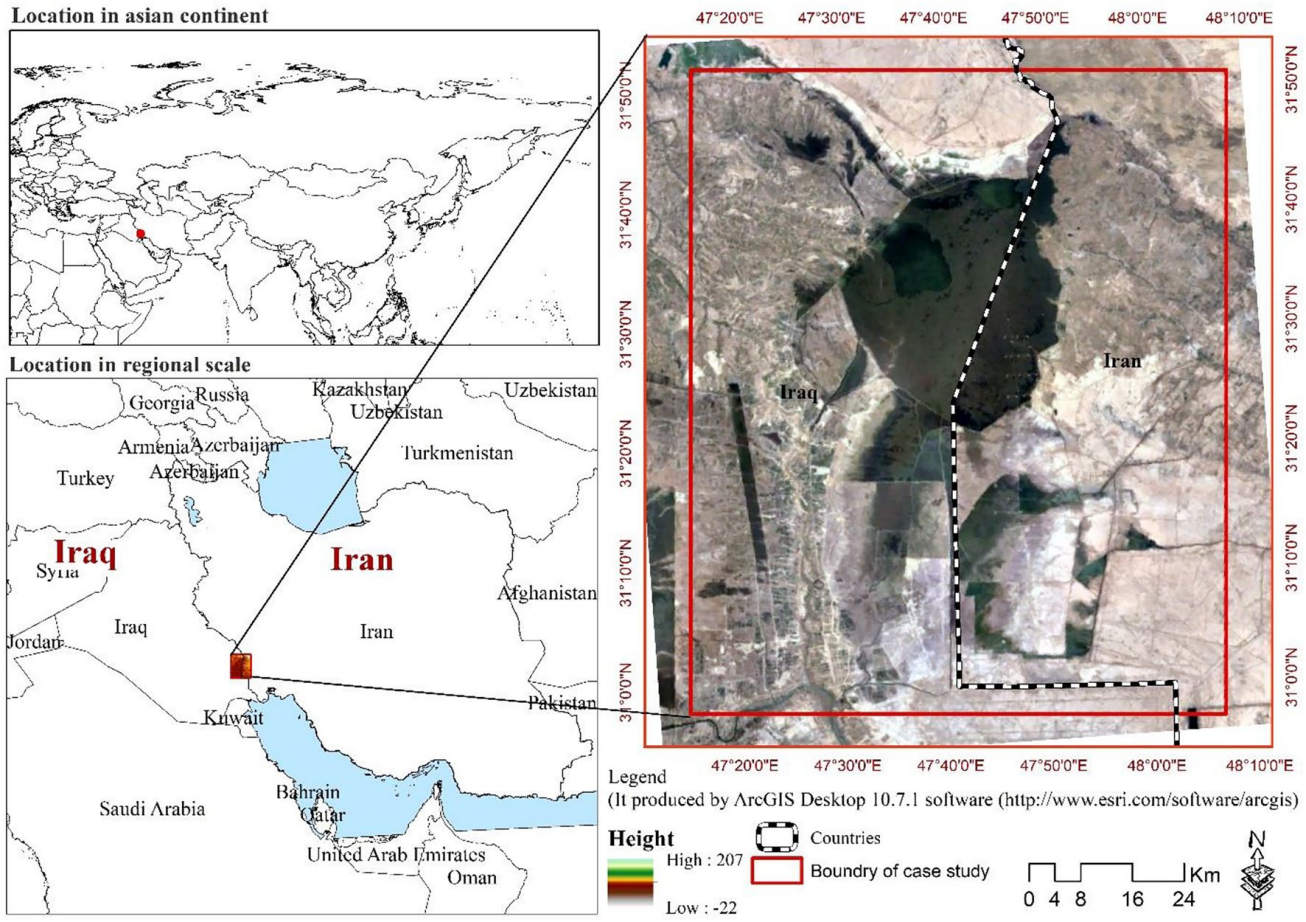
Geographical location of the study area.

Hoor Al-Azim wetland is a part of one of the largest wetlands in West Asia, called the Mesopotamian wetland, with an area of over 9000 km^2^. This wetland (Hor, marsh) is composed of the central Hor, Horalhammar (Hammar Marshes), and Horalhawizeh (Hawizeh Marshes). Central Hor and Horalhammar are entirely located in Iraq, and about two-thirds of Hawizeh is in Iraq and one-third is in Iran. The Iranian part is known as Hoor Al-Azim (Hadith^35,36^). Hoor Al-Azim wetland, with an area of 771,161 ha, is in the southwest of Khuzestan province, in the northwest of Bostan city, and near Chazabe village at the coordinates of 71° 51′ E and 71° 76′ W (Fig. 1). The Hoor Al-Azim wetland is a part of the permanent freshwater wetland in lower Mesopotamia, which is located in the borderline region between Iran and Iraq. The wetlands of Mesopotamia are one of the 200 most important biological areas in the world^5^. The Hoor Al-Azim wetland is important internationally for historical, cultural, and environmental reasons. However, it has always been directly and indirectly affected by anthropogenic degradation at the national and international levels. The primary threats to the water ecosystem from human activities, including dam construction in Turkey, extensive well drilling in Iraq, and the Karkheh Dam in Iran, all of which have significantly reduced the water levels of this vast ecosystem. Additionally, the degradation of the Hoor Al-Azim wetland is exacerbated by oil exploration, road and dyke construction, river deviation, and the aftermath of wars (Iran-Iraq and Gulf wars), alongside natural factors like recurring droughts^37^. The reduction in wetland area has directly contributed to vegetation loss and surface diminishment, leading to increased susceptibility to dust particles and the generation of fine dust^38^.

### Data preparation and mapping of modeling variables

Landsat satellite images from 1985, 2000, and 2015 produced by Nadaf^39^ were used to simulate land cover change dynamics in the Hoor Al-Azim wetland. During the pre-processing phase of satellite images, geometric, atmospheric, and radiometric error corrections were conducted. The fuzzy classification was utilized in the eCognition software to generate maps. Each classification class in eCognition is planned based on the class descriptions, and each description includes a set of fuzzy statements that allow the assessment of areas and logical functions. The output of this system has two parts as follows:

1. The fuzzy classification with partial information of classes provides a fragile classification for placing objects in a class.
2. The fuzzy principle can have individual conditions or a combination of several layers that include all evaluations for classes.

In eCognition, conditions are defined by statements that are entered for class descriptions. Definitions related to individual conditions, logical combinations of multiple conditions, and nearest neighbors are given below^39^.

**(a) Individual conditions:** Individual conditions are defined by a one-dimensional membership function. For visual object features, the one-dimensional membership function is defined by the graphical surface. Through the one-dimensional membership function, all available information about the relationship between areas and the assignment of classes can be completed.

**(b) Combined conditions:** in most cases, the principles required for land use classes cannot be described through individual conditions. Classes are often composed of combined conditions that are determined by the operator. In the area beyond, individual conditions can be expanded.

**(c) Nearest Neighbor:** Fuzzy perception from the point of view of the nearest neighbor, which is used in eCognition, automatically generates the multidimensional membership function. Five land use classes in the study area include humid land, water body, salt land, sparse vegetation, and dense vegetation (Table 1) (Nadaf et al. 2015).

**Table 1:** Land cover classification scheme.

Class name	Description
Sparse Vegetation	Low-density plant vegetation Includes *Phragmites australis*, *Carex brunnea*, and *Typha latifolia*
Dense vegetation	High-density plant vegetation Includes *Phoenix dactylifera*, *Ziziphus spina-christi*, *Prosopis cineraria*, and* Tamarix*
Water bodies	The area includes wetland and water bodies
Salt	The area includes salt and bare land
Humid land	The area includes regions with relatively high humidity and reed beds on the edge of the wetland

After assigning suitable weights to the image bands, segmentation was conducted. During this process, a scale parameter of 10 was selected, with homogeneity preferences set at 0.8 for color and 0.2 for shape, alongside 0.9 for shape smoothness and 0.1 for compactness.

The accuracy of the classified land cover maps was assessed using indices derived from the error matrix^40^. To enhance the comprehensiveness of the study area's coverage, ground truth points along with their land cover data were meticulously gathered. Each sample site was precisely marked with GPS coordinates and corroborated with ground truth information. The accuracy of the classified maps was rigorously evaluated by comparing the results of classified map data against the ground truth on a per-category basis, utilizing an error matrix to delineate the classification results' reliability. Accuracy assessment of the classified maps was done based on the error matrix and Kappa Statistic with about 250 random samples of ground truth data for each year^41^. Kappa was calculated using Eqs. (1)^42^.

1 $$K= \frac{N\sum_{i=1}^{r}{X}_{ii}-\sum_{i=1}^{r}{X}_{i+}{ X}_{+i}}{{N}^{2}-\sum_{i=1}^{r}{X}_{i+ }{X}_{+i}}$$

where *r* is no. of rows in the error matrix, *X*_*ii*_ is no. of observations in row i and column i, *X*_*i*+_ is total observation in row i and *X*_+*i*_ is total observation in column i.

## Variables

Slope, digital elevation model, distance from main roads, distance from the river, distance from sparse vegetation, distance from dense vegetation, distance from humid lands, distance from water bodies, distance from salt lands, evidence likelihood to change the map, annual average temperature, and precipitation variables (Supplementary Table 1) were produced to select effective variables in the expression of changes in Hoor Al-Azim wetland (Supplementary Fig. 1). Topography has been considered by other studies as an effective driver variable determining land use and land cover changes^43,44^. Distance from water bodies and vegetation were important factors in modeling changes in the Saudi Arabian eastern coastal city of Dammam^45^. Distance to the rivers was recognized as an essential underlying driving force of land cover change in Iranian Northern Zagros forests^46^. Proximity to main roads and evidence likelihood to change map have been reported as essential variables in modeling the dynamics of land cover in the Zanjan Province, Iran^19^. The total annual precipitation is considered an explanatory variable in the generation of land use scenarios in the Catamayo Chira basin, located between Ecuador and Perú^11^.

The digital elevation model of the studied area, with a spatial resolution of 30 m, was extracted from the ASTER digital elevation model. The slope layer was also produced using a digital elevation model map. Sparse vegetation, dense vegetation, and rangeland were extracted from the land cover image of the older year (in the model input) as Boolean layers, and the variable representing the distance from each of these classes was prepared^47^. The evidence likelihood to change map was generated using the evidence likelihood transformation tool. In this way, the map of changes during the study period was produced as a Boolean layer and placed in the Transition or land cover layer section. Also, the land cover map of 1985 (a map of an older year in the model's input) was entered into the variable input section of the transformation panel, and the evidence likelihood to change map was generated.

## Research method

**Figure 2:**
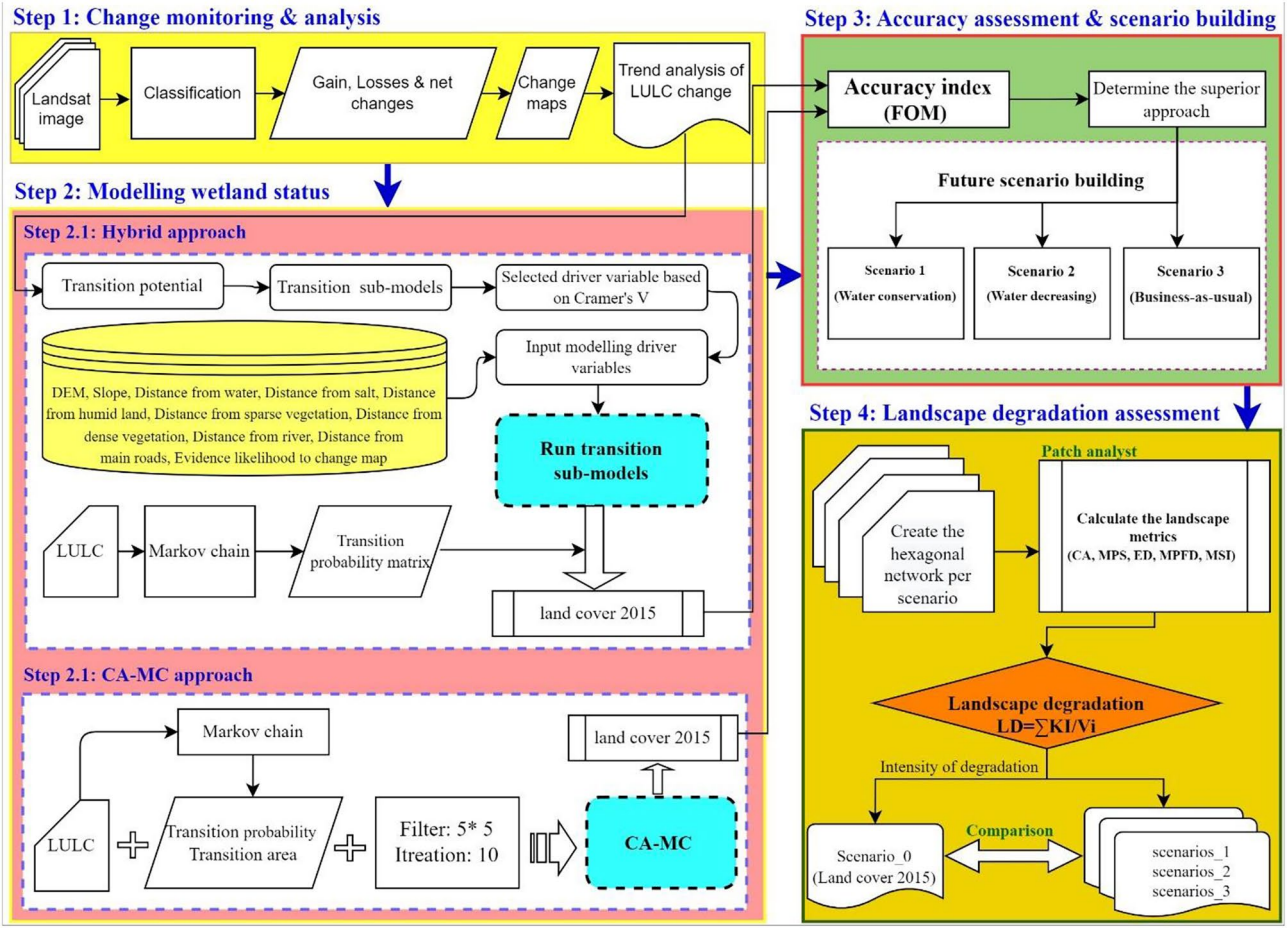
Research method.

This research was done in four steps, according to Fig. 2.

### Step 1: Monitoring and analysis of land cover changes

The analysis of changes in the study area was calculated using LCM for the first period (1985–2000) and the second period (2000–2015). LCM can determine the change process based on location, calculate transition probabilities between land cover classes, and predict future land cover maps^44^. These changes include gains, losses, and net changes for each land cover class and the transition from one class to another. Also, maps of the spatial trend of changes from water bodies to other land cover in Hoor Al-Azim International Wetland were produced using the results obtained from the analysis of changes.

### Step 2: Modeling of the wetland status

#### Hybrid approach

The present study integrates the approaches of the artificial neural network, Markov chain, and multi-objective optimization into a single model. LCM uses transition potential maps for each transition sub-model of land cover classes to assign simulated transitions based on land cover changes in the past^46^. In the present study, a multi-layer perceptron artificial neural network was used to model the transition potential. The network uses feed-forward algorithms to calculate weights for input values, input layer nodes, hidden layer nodes, and output layer nodes, which are transferred to the hidden layer and the output layer^48^. In this study, the number of repetitions is set at 10,000 to achieve high accuracy. Accuracy rate statistics and Skill statistics are also used to evaluate the neural network. The value of the Momentum Factor in the present study was considered to be 0.5. The LCM model uses Cramer's V to examine the relationship between each variable, the distribution of land cover classes, and the changes that have occurred. The value of this coefficient is between 0 (no correlation) and 1 (complete correlation)^49^, which is calculated using Eq. (2).

2 $$V= \sqrt{\frac{{X}^{2}/n}{\text{min }(k-1, r-1)}}$$

where *X*^*2*^ is the chi-square statistic, *n* is the number of samples, and* k* and *r* are the number of rows and columns in the probability table, respectively^47^.

After determining the power of the explanatory variables using the Cramer coefficient (a high Cramer coefficient means that the explanatory potential of the variable is good), ten variables were considered, and the artificial neural network with slope variables, digital elevation model, distance from main roads, distance from the river, distance from sparse vegetation, distance from dense vegetation, distance from humid lands, distance from water bodies, distance from salt lands, and the evidence likelihood to change map was instructed.

In order to ensure the selection of the best sub-models for modeling, a spatial analysis was performed on the change pixels using explanatory variables of the changes^50^. The sub-models used in the research include the water body to sparse vegetation, water to dense vegetation, water to humid land, water to salt land, humid land to sparse vegetation, dense vegetation to sparse vegetation, dense vegetation to humid lands, and salt land to humid lands.

The results of the artificial neural network are combined with Markov chain and multi-objective land allocation to simulate the spatio-temporal dynamics of land cover changes in the Hoor Al-Azim wetland. In this study, the Markov chain model was used to estimate the rate of change that can occur in a specific future year based on previous land cover maps and the probability of change from one land cover class to another. A prediction map for 2015 was created using the Markov chain to evaluate the accuracy of the model^51^. After ensuring the accuracy of the model, the land cover maps for the years 2000 and 2015 were used to predict the land cover maps for 2030^44^.

#### CA-MC approach

The CA-MC model is a combination of cellular automata, Markov chain, and multi-objective land allocation, which are used to predict future changes in land use and land cover^52,53^. In this research, the CA-MC approach was used in 1985 and 2000 to predict land cover changes in 2015. The basic assumption of the approach is that the land cover status in the future (*t* + 1) can be defined as a function of the current land cover (*t*) as shown in Eq. (3), where ${x}_{(\text{t}+1)}$ shows land cover at time *t* + 1 and *x* shows land cover at time *t*^54^. The structure of the Markov chain model for modeling land cover changes includes a vector (*N*) with dimensions $M\text{x} 1$(M represents the number of land cover classes) that describes the current land cover distribution and a $M \text{x} M$ matrix of transition probability (*P*) between each pair of land cover classes *i* and *j*. The transition probability is calculated according to Eq. (4).

3 $${x}_{(\text{t}+1)}={f}_{(\text{xt})}$$

4 $$\sum_{j=1}^{m}{p}_{ij}=1 i =\text{1,2},3\dots m$$

### Step3: Accuracy assessment and future scenario planning

The prediction error and accuracy of the model were evaluated based on three land cover maps: the actual maps from 2000, and 2015, and the map generated from the 2015 model simulations. The FOM index was calculated according to Eq. (5) to evaluate its accuracy. The value of this index is between 0 and 100, where 100 indicates a complete overlap between the ground truth map and the simulated map^55^.

5 $${\text{FOM}} = {\text{Hits}}/\left( {{\text{Hits}} + {\text{Misses}} + {\text{False}}\;{\text{Alarm}}} \right)$$

where Hit (the model predicts the change between 2000 and 2015 and the ground truth map also shows the change from 2000 to 2015), Miss (the model did not predict any change between 2000 and 2015 while the ground truth map has changed), and False Alarm (the model predicts the change during the years 2000 to 2015 while the ground truth map shows no change) are included.

## Scenario creation

Three scenarios were considered for modeling land cover changes in 2030, and the labels and selected sub-models for each are described below.

*Scenario 1 (Water Conservation)* According to the sixth development plan (2016–2021)^56^ in terms of social, economic, and environmental sustainability in Iran, we selected the sub-models in order to increase the transition probability of other classes to water. In this scenario, the sub-models used include sparse vegetation to water, dense vegetation to water, humid lands to water, and salt lands to water.

*Scenario 2 (Water Decreasing)* There is a risk of the wetland drying up due to upstream dams of the wetland, oil facilities construction, new roads development, and water exploitation^57^. This scenario assumes high economic growth and predicts continued shifts in consumption patterns. In this scenario, the sub-models used include water body to sparse vegetation, water to dense vegetation, water to humid lands, and water to salt lands.

*Scenario 3 (Business-as-Usual)* This scenario assumes current trends and projects the wetland's future status for 2030. It is selected to explore the sensitivity to recent drivers of changes using sub-models such as water to sparse vegetation, dense vegetation, humid lands, and salt lands; humid land to sparse vegetation; dense vegetation to sparse vegetation, humid land; and salt lands to humid lands.

### Step 4: Evaluation of the landscape degradation

To assess the degradation of the landscape, the study area was divided into a network of uniform hexagonal units. Hexagons are the closest regular geometric shapes to the circle, which can cover an area with no overlap and have a smaller perimeter than a square with the same area, so it reduces the error caused by the edge effect^58^. In this study, due to the large scope of the study^59^ and the quality and accuracy of the data^60^, hexagons with an area of 40,000 hectares were selected as management and monitoring units after numerous tests and visual checks to ensure that all changes were included. Management and monitoring units should be large enough to represent different types of land cover^61^ and to reflect the severity of degradation caused by land cover change and landscape metrics in temporal and spatial gradients. The quantification of CA, MPS, ED, MPFD, and MSI metrics was conducted to evaluate the degradation of the landscape in the study area using the Patch Analyst tool, and the details of the metrics are presented in Table 2. The application of landscape metrics and the scientific basis of their relationship with land degradation is a precedent in various studies^32,30,31,59,62^. In this research, the severity of degradation is a relative value obtained from the value of the metrics that influence landscape degradation. These metrics are a measurement category of interval scales have converted into ordinal scales during mathematical processing. In each of the hexagon units, the rate of degradation was calculated based on Eq. (6).

6 $$LD=\frac{\sum KI}{{V}_{i}}$$

where $\sum KI$ determines the intensity of activities in the study unit, K represents the degradation intensity class of each metric, *I* is the metric, and V_i_ represents the intensity of ecological vulnerability. In this research, the value of *K*, which represents the degradation intensity class of each metric, has been obtained after calculating the landscape metrics and normalizing them based on the fuzzy logic in Eq. (7).

**Table 2:** Definition and characteristics of landscape metrics calculated in the research^63^.

Landscape metrics	Representation	Equation	Range	Units
Class area (CA)	Sum of the areas of all patches	$\sum_{j=1}^{a}{a}_{ij}(\frac{1}{\text{100,000}})$	CA ≥ 0, without limit	Hectares
Mean Patch Size (MPS)	Average patch size	$\frac{\sum_{\text{j}=1}^{\text{n}}{p}_{ij}}{{\text{n}}_{\text{i}}}$	MPS > 0, without limit	Hectares
Edge Density (ED)	Amount of edge relative to the landscape area	$\frac{\sum_{k=1}^{m}{e}_{ik}}{\text{A}} (\text{10,000})$	ED ≥ 0, without limit	Meters per hectare
Mean Patch Fractal Dimension (MPFD)	Shape Complexity	$\frac{2\text{ln}(0.25{p}_{ij})}{{\text{ln}a}_{\text{ij}}}$	1 ≤ MPFD ≤ 2	None
Mean Shape Index (MSI)	Shape Complexity	$\frac{{\sum p}_{ij}}{{\text{min}p}_{\text{ij}}}$	MSI ≥ 1, without limit	Meters

7 $${Z}_{i}=\frac{{x}_{i}-\text{min}(x)}{\text{max}(x)-\text{min}(x)}$$

In Eq. (7), Z_i_ represents the normalized value of the metric in the unit of hexagon *i*.

X_i_ is the metric quantity in hexagon units *i*. The $\text{min}(x)$ indicates the minimum metric quantity between all the units of the hexagonal network. Degradation severity codes were assigned based on the impact of suitable and unsuitable land covers on the wetland landscape and the effect of metrics, as shown in Table 3.

**Table 3:** Coding of the intensity of degradation of land covers in the region according to the calculated landscape metrics.

Land cover/ metric	Group metrics 1 (CA, MPS)		Group metrics 2 (ED, MPFD, MSI)	
	Code	Rang of Zi	Code	Rang of Zi
Unsuitable Land cover (Sparse vegetation, Salt)	1	0–0.25	1	0.75–1
	2	0.25–0.5	2	0.5–0.75
	3	0.5–0.75	3	0.25–0.5
	4	0.75–1	4	0–0.25
Suitable Land cover (Water, Humid land, Dense vegetation)	1	0.75–1	1	0–0.25
	2	0.5–0.75	2	0.25–0.5
	3	0.25–0.5	3	0.5–0.75
	4	0–0.25	4	0.75–1

The land cover degradation index was qualitatively determined between 0 and 1 by expert opinion. Lower values of this index indicate less degradation, and vice versa (water: 0, humid lands: 0.1, dense vegetation: 0.2, sparse vegetation: 0.7, and salt lands: 1). Vulnerability refers to the degree to which a system, subsystem, or system components are susceptible, unable to cope with its adverse effects, and are damaged when exposed to stimulating factors (disturbance or pressure)^64^. The intensity of ecological vulnerability was also calculated based on the percentage of suitable land cover in each hexagonal unit.

## Results

### Analysis of changes in land covers

The dataset used for modeling the wetland landscape dynamics includes variables and land cover maps from the years 1985, 2000, and 2015. These maps were meticulously prepared using GIS technology and are illustrated in Supplementary Figs. 1 and 2*.* An overall Kappa coefficient of 0.8546, 0.9110, and 0.8887 was obtained for the Landsat images of 1985, 2000, and 2015, respectively. The detailed results of the confusion error matrix, including errors of commission, omission, and Kappa Statistics in each class, are presented in Supplementary Table 2.

**Figure 3:**
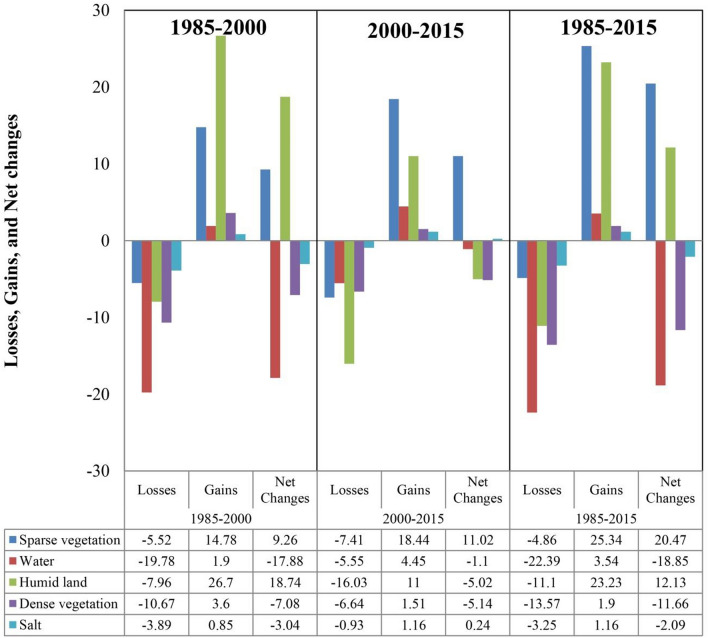
Gains, losses, and net change in each land cover class (% of the area) during 1985–2000, 2000–2015, and 1985–2015.

The water bodies accounted for 25.1% of the total area in 1985. By 2000, this percentage has decreased by 7.1%, resulting in an annual reduction rate of 8.3%. The gain and loss in water bodies were 15,735 ha and − 163,487 ha, respectively (Fig. 3). From 2000 to 2015, the net change from dense vegetation, water, and salt lands to sparse vegetation was 18,034, 9985, and 62,946 ha, respectively. The area of water bodies shrank by 9,107 hectares, corresponding to an annual reduction rate of 1.1%. Sparse vegetation increased during this period at an annual rate of 2.5% and 91,509 ha (Supplementary Table 4). The net change from water to humid and salt lands was 9,741 and 7,449 ha, respectively. According to the spatial trend map (Supplementary Fig. 3), the most significant changes from water to salt lands occurred in the southern region during 1985–2000 and in the centeral and southern parts of the studied region during 2000–2015.

### Modeling the transition potential and hybrid approach

**Figure 4:**
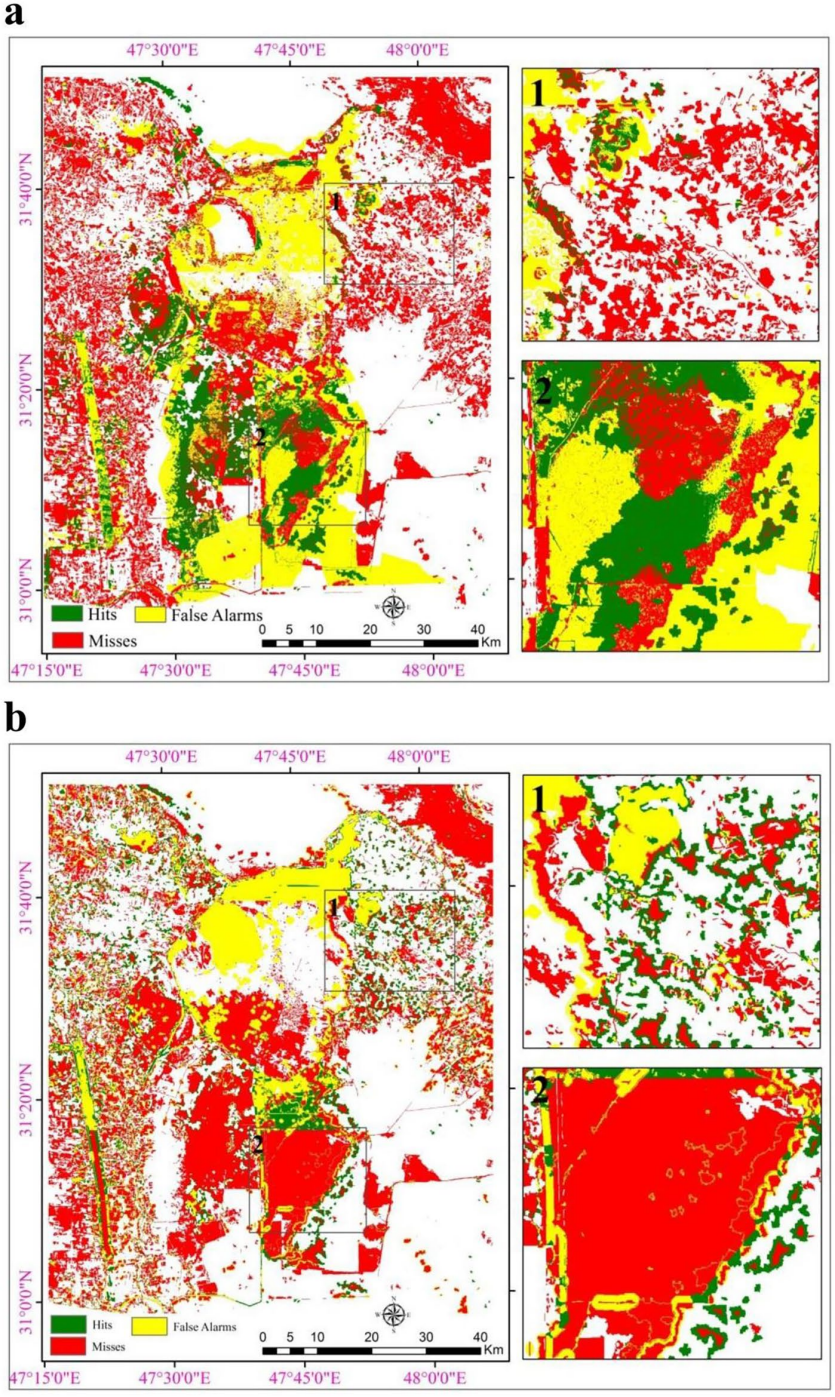
Modeled land cover maps of 2015 using hybrid (**a**), CA-MC approaches (**b**), and Hit, Miss, and False Alarm areas in evaluating the accuracy of the modeled maps.

The importance of the variable in the artificial neural network model was measured using Cramer's coefficient, which ranges from 0 to 1. According to Cramer's coefficients in Table 4, all variables showed "good" power in describing changes. According to Supplementary Table (3), the water to salt lands sub-model showed the highest accuracy rate (98.04%), and the dense to sparse vegetation sub-model showed the lowest accuracy rate (82.22%). According to the results of the Markov chain, the water with the highest probability will turn into humid lands from 2015 to 2030 (0.3633). The map modeled by the developed hybrid approach is depicted in Fig. 4a.

**Table 4:** Results of Cramer's coefficients between variables and land cover classes.

Variable	Water	Humid land	Sparse vegetation	Dense vegetation	Salt	Overall V
Slope	0.4190	0.2031	0.1067	0.0795	0.0153	0.2213
DEM	0.1933	0.1977	0.2313	0.2464	0.0469	0.1923
Distance from main roads	0.1173	0.2014	0.2287	0.3554	0.0528	0.2141
Distance from river	0.1757	0.2773	0.2845	0.1591	0.0820	0.1960
Distance from sparse vegetation	0.2331	0.3490	0.4965	0.4802	0.0640	0.3524
Distance from dense vegetation	0.1718	0.4355	0.1993	0.4766	0.0933	0.2959
Distance from humid land	0.3787	0.3401	0.2459	0.4847	0.1025	0.3340
Distance from water	0.2898	0.2988	0.1654	0.3051	0.0900	0.2389
Distance from salt	0.2396	0.3446	0.1492	0.3104	0.0915	0.2326
Evidence likelihood to change map	0.3358	0.4353	0.3856	0.5149	0.1139	0.3698
Annual average precipitation	0.1896	0.2672	0.2585	0.3054	0.0897	0.2262
Annual average temperature	0.1709	0.4631	0.2574	0.6581	0.0682	0.3729

### Modeling changes with the CA-MC approach

The changes in land cover area were obtained using Markov chain analysis between 1985 and 2000, based on the area transition matrix in Table 5. Finally, the CA-MC approach was implemented using the area transition matrix. Land cover maps were repeated 10 iterations with a 5 × 5 filter, and the land cover change map for 2015 was depicted as Fig. 4b.

**Table 5:** The matrix of resulting area transition obtained in the CA-MC approach.

Land cover	Sparse vegetation	Water	Humid land	Dense vegetation	Salt
Sparse vegetation	1,237,843	15,816	854,004	86,438	7547
Water	96,349	124,925	341,910	77,682	18,411
Humid land	1,439,380	1302	3,391,711	40,659	22,543
Dense vegetation	224,941	116,193	345,128	492,394	295
Salt	2128	0	81,560	230	1600

### Assessing the accuracy of modeled maps

The FOM index range, derived from evaluating the accuracy of the hybrid model, is 15.6%. This value signifies the high efficiency and capability of the model utilized in this wetland, attributed to its substantial size and the varied changes during the calibration period in contrast to the simulation period. Also, the accuracy of the CA-MC model was estimated to be 14.42% (Fig. 4). The Hits, Misses, and False Alarms zones are shown in Fig. 4.

### Scenario planning of the future status of the wetland

**Figure 5:**
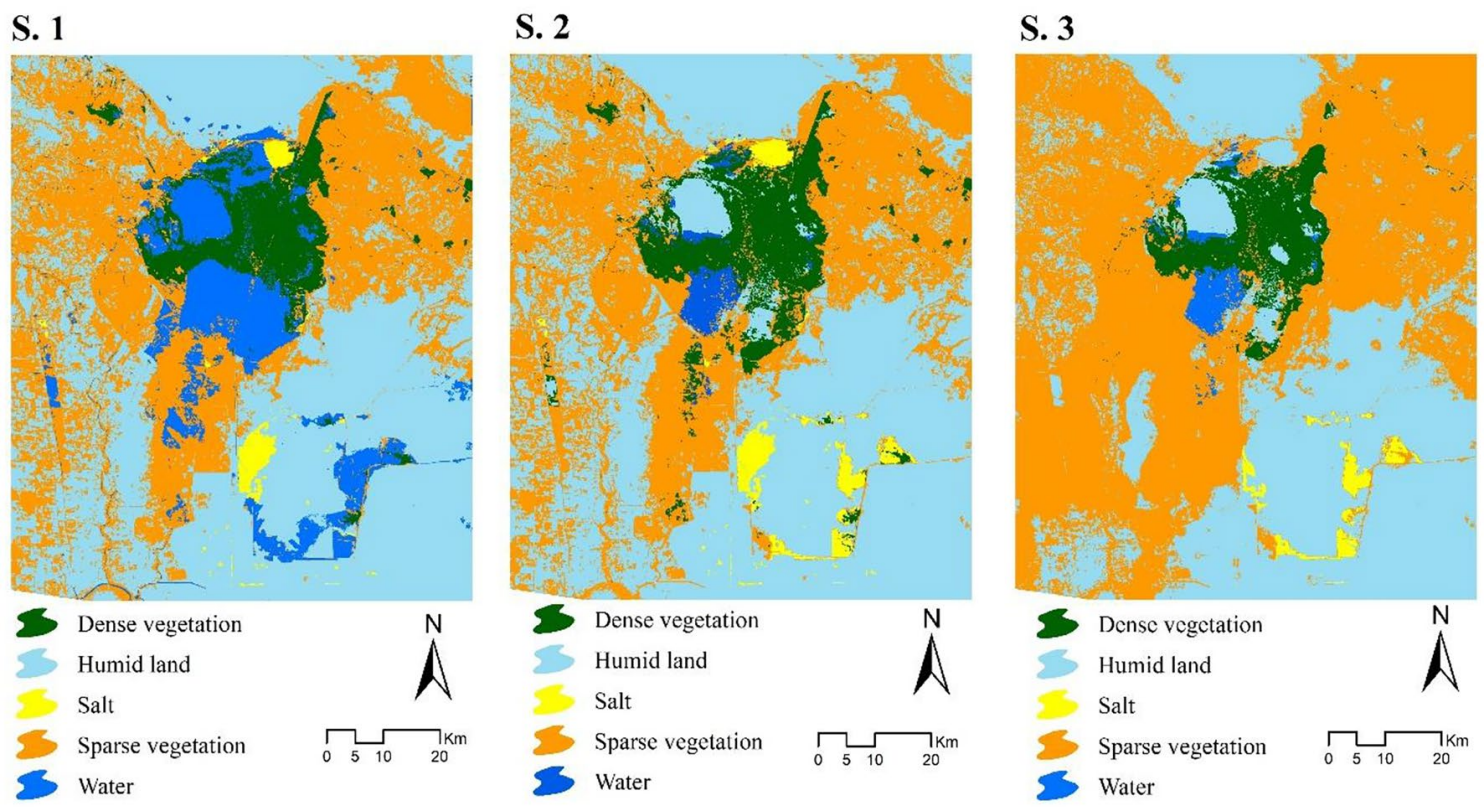
Simulated scenarios of Hoor Al-Azim International Wetland in 2030.

After evaluating the accuracy of the model, different land cover scenarios for 2030 were produced based on the three policies outlined in the methodology, utilizing the superior hybrid approach identified (Fig. 5).

### Evaluation of the landscape degradation

**Figure 6:**
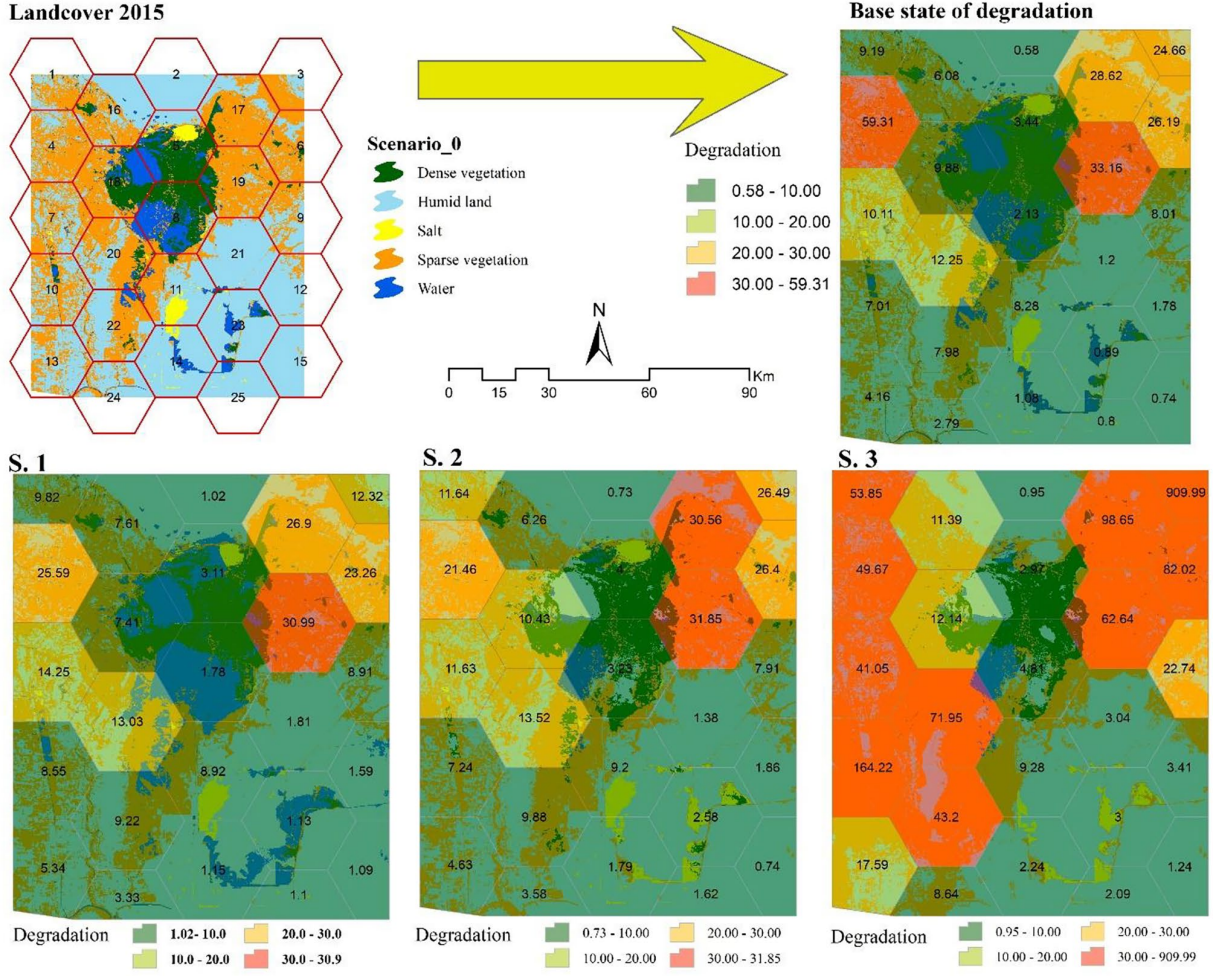
Degradation gradient in the hexagonal units of the wetland landscape in three simulated scenarios compared to the base scenario.

In Fig. 6, the hexagonal network consisting of 25 units is depicated as the basis for evaluating degradation in the wetland. This map was created by overlaying the land cover map with the hexagonal network. Additionally, a desired network was developed for future scenarios. The rate of landscape degradation was calculated using to the mentioned method based on landscape metrics in Excel software (Eq. 3). As shown in this figure, the baseline scenario includes high degradation in some hexagonal units. However, the majority of its units experienced a degradation degree of less than 10 in the base year of 2015. For a more effective comparison, the classification of degradation degrees for both the baseline and future scenarios was categorized into four relative groups. This representation illustrates those scenarios 1, 2, and 3 are projected to experience increasing levels of degradation in the future. The degradation rate per hexagonal unit is labeled.

**Figure 7:**
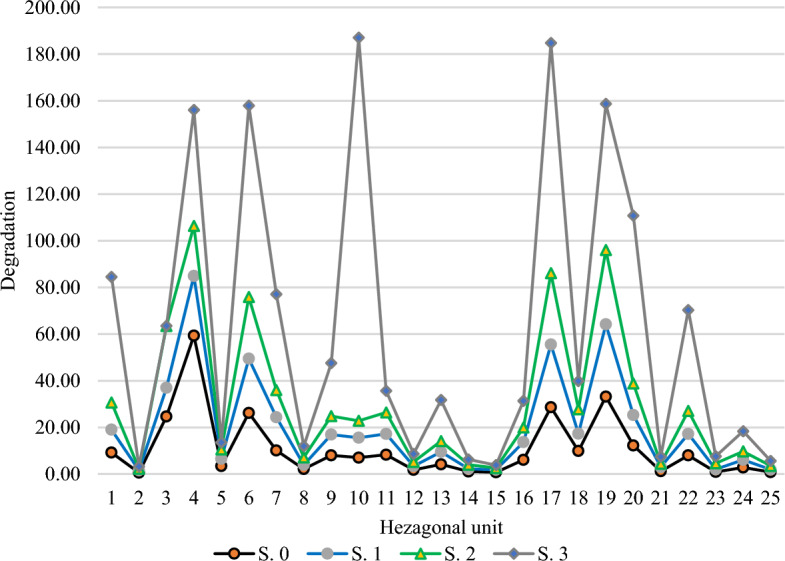
Comparison of the rate of degradation in the study units in the scenarios.

The comparison of the degradation rate in hexagonal units for each of the baseline and future scenarios is shown in Fig. 7. In general, the sum of degradation rate of hexagonal units from the base situation of 2015 with a value of 270.33 will reach 229.23, 250.60, and 1682.80 in scenarios 1, 2, and 3, respectively.

## Discussion

### Patterns of landscape change

Examining the images of 1985–2000, 2000–2015, and 1985–2015 reveals significant changes in the land cover classes of Hoor Al-Azim wetland and its surroundings, especially in the water level. The decrease in the wetland's water level has resulted in changes in other covers, like dense and sparse vegetation. Throughout these periods, the wetland has undergone significant degradation, as evidenced by a marked reduction in water bodies from 207,511 ha in 1985 to 50,264 ha, indicating a decreasing trend. Additionally, the area of salt lands decreased from 33,181 ha in 1985 to 9628 ha in 2015 due to receding water and the growth of vegetation, such as reeds and cholan, along the wetland's shore.

Consistent with findings from studies such as those by Yu et al.^65^ and Zhu & Gong^66^, other wetland regions have similarly experienced a reduction in water, with human activity identified as the main threat to these ecosystems. Conversely, the wetland area has increased in the Abitibi-Témiscamingue region of Canada, attributed to the minimal impact of human activities in this area^44^.

Various factors in the anthropogenic process have led to changes in the Hoor Al-Azim wetland’s ecosystem. The main source of water for Hor comes from its upstream rivers, including the Karkheh in Iran and the Tigris and Euphrates in Iraq. Any interference, whether human or natural, with the flow rate of these rivers will significantly impact the water requirements of the wetland. In Iran, the Karkheh River and its branches supply most of the water to the Hoor Al-Azim wetland^39^. The construction of the Karkheh Dam in 1997 and its impoundment during 1998–2002 were of the most important factors in reducing the level of the wetland in such a way that the flow entering the wetland through the Karkheh River was 5450 million m^3^ annually on average before the construction of the dam, which has decreased to 4700 million m^3^ since 1998–2002. The evaporation rate of the wetland is 1.9 million m^3^ per km^3^^67^. Human factors, including resource threat and pollution since the war between Iran and Iraq, have degraded the Hoor Al-Azim wetland^68^. Factors like road construction, oil drilling, lack of limitations, and loss of effective protection have led to its drying and turn it into an area with dense, sparse vegetation or salt land. The southern part has been affected by the Azadegan oil field development plan, while operations in the northern part are still ongoing^5^. Key strategies for wetland restoration include preventing habitat fragmentation by avoiding road and dike construction, harnessing local community support for wetland conservation and ecotourism, and utilizing national and international funding to provide protective infrastructure. Additionally, strategic zoning can optimize resource allocation for agriculture and ecotourism.

### Dynamics modeling of wetland

The success and performance of simulation models based solely on quantity cannot provide researchers with information on spatial criteria and morphological features. Therefore, a spatially explicit scheme is needed to evaluate the performance of spatially explicit predictive models. In addition, this plan is expected to offer insight into the model's behavior. Therefore, this research proposed an advanced method based on the study of the landscape pattern to evaluate the model's performance^26^. According to the characteristics of the studied area, the hybrid approach used in this study was beneficial for comprehending the intricate dynamics of the landscape pattern of the Hoor Al-Azim wetland in the region. The MOLA algorithm used by LCM was successfully applied in the dynamic modeling of wetland changes. This study also evaluated the comparative simulation capabilities of integrated MLP_Markov and CA_Markov models in terms of ecology using the FOM index. Using this index is one of the most valuable and accurate methods in comparing and confirming the accuracy of simulation models, and it has been used in numerous studies. Based on the results of the accuracy evaluation, the FOM index value in the current study was higher for the MLP_Markov model than for the CA_Markov model. The rate of this index was found to be 14.73% in the comparison between the ground truth map and the simulated map of 2015 in Tianjin, China^69^. In the study of Zabihi et al.^70^, this index was reported to be 10.53% in the Talar watershed of Iran, which indicates that the accuracy of modeling in the present study is higher than those in the two mentioned studies.Kourosh Niya et al.^27^ used an adding/deleting approach to increase the accuracy of hybrid models and assessed the accuracy of different models using the FOM index in Iran's Qeshm Island. The value of the FOM index when using this approach was 7.8, which was better than when not using it. Shoyama et al.^71^ developed high-resolution land use maps by utilizing a GIS-based vegetation database. They also created scenarios to project potential land changes in Japan by 2050 on a national scale. The value of the FOM index in that study was 0.58%, which is significantly lower than its value in the present study.

### Estimating the severity of degradation

The study generated different scenarios for 2030 and evaluated their impact on the landscape comparison to the baseline situation in 2015. By utilizing a degradation model and a network of hexagonal units, landscape metrics were used to estimate the different conditions resulting from each policy adopted.

In a study conducted in East India, researchers found that landscape criteria and spatial characteristics of land use/land cover are effective tools for identifying land fragmentation and degradation^72^. Their analysis of the Chatra wetland revealed significant deterioration, with a 60% degradation over 28 years, due to urban construction. The transformation and change of natural land cover around the wetland have a destructive impact on the wetland ecosystems, mirroring the results of this research. Although, the education and social learning of people as stakeholders of land use change have been recognized as effective in improving resilience under drought conditions in Iran^73,74^.

While numerous studies have analyzed historical degradation rates in wetland ecosystems, there has been limited research projecting degradation into future scenarios.Hu et al.^8^, provided a framework for extracting the drivers of wetland degradation and found significant differences in degradation rates across scenarios, similar to the findings of this research. Evaluating wetland degradation has been a critical issue, and Cui et al.^6^ proposed a framework that showed rapid ecological degradation in the Yancheng National Natural Reserve wetland in China over 30 years. The innovative approach used in this study, combining landscape metrics and land cover dynamics, offers a new method for evaluating future wetland degradation. The severity of degradation in management scenarios can be influenced by land managers, who can change metrics by altering land covers. Suitable land covers, such as water bodies, humid land, and dense vegetation (with increased values of CA and MPS metrics) contribute to lower degradation, while unsuitable land covers like salty lands and sparse vegetation (with decreased values of these metrics), lead to higher degradation. Also, less degradation caused by the decrease in ED, MPFD, and MSI values in suitable land covers and an increase in the value of these metrics in unsuitable land covers. On the other hand, the high percentage of water, humid land, dense vegetation and low percentage of salty lands and sparse vegetation in hexagonal units reduces the severity of degradation.

Awareness of different strategies and their consequences in various scenarios is crucial for understanding the future situation of a region. Territorial managers and custodians can avoid unfavorable paths and move towards a more favorable future by adjusting their strategies. Simulation results of scenario 1, an optimistic scenario, show that water conservation and preventing the conversion of water bodies improve or minimize degradation compared to the baseline situation. Preserving water requirements, preventing drilling and oil exploration, and dredging waterways are important considerations for achieving this goal. In the intermediate scenario, the rate of degradation also decreases. If the current historical scenario trend continues, the degradation situation will worsen significantly by 2030. Protective measures have been taken for sustainable wetland utilization and conservation in Iran and Iraq. Three units for cooperation and monitoring, education and awareness, and science and technology are working to implement the Ramsar Convention on Hoor Al-Azim Wetlands (Iran) and Horalhawizeh (Iraq). They tried to develop a mechanism for effective communication between the Center for Restoration of Marshes and Wetlands and oil companies to mitigate the impact on the wetland.

Change in Hoor Al-Azim wetland is a dynamic process, and uncertainties can affect its trajectory of wetland. The model was utilized under the assumption that the explanatory drivers of wetland loss will not vary significantly within the next 15 years^75^. The exclusion of highly dynamic variables such as roads may affect the outcomes of the model. It is proposed that new roads will be added to the model as dynamic input. For the utilization of precipitation and temperature data, maps at a 1 km resolution were employed, despite the lack of availability in the desired scale. This may introduce a degree of uncertainty in the model's input due to the scale of these maps. For future studies, it is necessary to examine the changes in government policies and economic development in the region that can affect the results of the transition potential modeling. The accuracy of the simulated land cover maps is strongly related to the accuracy of input data layers for the model, such as historical land cover maps. Temporal and spatial heterogeneity make wetlands hard to map with remote sensing imageries. The coarse classification of wetlands may also introduce bias, as Hoor Al-Azim wetland is a permanent freshwater wetland surrounded by reed that may overlap with sparse or dense vegetation classes. A finer and more accurate classification of wetlands is needed to capture them. The accuracy of classified land cover maps extracted from remotely sensed data with moderate spatial resolution of the Landsat images can lead to lower reliability than that of high-resolution satellite images in simulated maps. Therefore, it is suggested to use sensors with higher spatial resolution than Landsat images to produce more accurate land cover maps. The scientific findings of the current research can also be used as input in studies analyzing the impact of spatio-temporal future land cover scenarios on ecosystem health assessment. The study of determining the water requirement of the wetland under the impacts of different future land cover scenarios and providing optimal solutions for supplying the water demands of the wetland should be investigated in the future.

## Conclusion

The present study aims to develop a highly accurate model for simulating future scenarios of land cover changes in Hoor Al-Azim International Wetland. The methodological approach employed involves a comparative analysis between the hybrid model—integrating an artificial neural network, Markov chain, and multi-objective optimization—and the cellular automata-Markov chain model. It was hypothesized that the LCM model would have a higher transition accuracy potential than the CA-MC model due to the use of artificial neural networks in modeling. The model-generated maps underwent successful validation via the FOM index application. The LCM model allowed us to generate three scenarios for 2030 concerning achieving higher accuracy. Scenario 1 (Water Conservation) will include the largest percentage of water bodies in the future. During 1985–2015, a decreasing trend was seen in the water bodies, humid lands, and dense vegetation, while sparse vegetation and salt lands demonstrated an increase in Hoor Al-Azim wetland. Evaluation of degradation in hexagonal units also showed the least degradation in the Water Conservation scenario compared with the other two scenarios in 2030. Therefore, appropriate and timely management measures and planning should be done by decision-makers to create sustainable development and protection of the wetland, along with environmental protection strategies, to reduce the intensity of changes.

We applied the Cramer's V coefficient, a chi-square-based metric of association between two variables, to quantify the significance of each variable in elucidating changes in the wetland. The results of Cramer's coefficient, which indicates the relationship between explanatory variables and land cover classes, showed that the DEM with a total Cramer's coefficient of 0.1923 had the least impact on the changes of Hoor Al-Azim wetland, while annual average temperature (0.3729) indicated the greatest impact on the changes of the region. Given that temperature and precipitation significantly influence the dynamics of the Hoor Al-Azim wetland, it is crucial to assess the effects of projected climate change scenarios and human-induced activities on the wetland's future state, with particular emphasis on water yield and water requirement. The accuracy rate of modeling results using artificial neural networks in different sub-models was also obtained between 0.64 and 0.96, demonstrating its high ability to model transition potential. Although the simulation results indicated a decreasing trend at the wetland water level, there are still more considerations for future modeling studies. For example, access to better spatial resolution in satellite imagery provides more detailed information about different class types that may not be detected by LANDSAT-based classification.

Rapid and extensive land cover changes in Hoor Al-Azim International Wetland might have serious environmental effects. For example, changes in the hydrologic regime and potential biological responses may cause changes in habitat quality and the abundance of native species and wintering birds in the wetland. To enhance the restoration and ongoing conservation of the wetland, practical measures could include curbing livestock overgrazing, raising awareness of the wetland's diverse benefits to ensure its protection, engaging local communities as environmental stewards, establishing expert-led monitoring and evaluation programs, and forming a protective buffer zone around the wetland by environmental agencies to thwart harmful human encroachment. Additionally, legal enforcement can be utilized to limit the spread of oil company operations and prevent the discharge of oil and urban wastewater into the wetland, coupled with the establishment of a crisis management center by Iran and Iraq. It is suggested to model water yield and sediment retention ecosystem services under the influence of land cover changes in subsequent research studies. Also, the change in the temperature and precipitation pattern under future greenhouse gas release scenarios should be modeled and added as a factor in the modeling of land cover changes. Also, using the results and method applied in this research can warn decision-makers and territorial managers about the process of anthropogenic changes and help them take action, plan, and sustainably protect the wetland to reduce the intensity of changes that lead to degradation. In general, the developed process can act as a decision-support system for managers and decision-makers. In this way, managers can understand the degradation effects of management plan alternatives in the units of study area before implementation with the help of the LD index calculation. Another use of our result in management is targeting a certain range of the degradation index according to the time and financial limitations of any management plan alternatives.

### Supplementary Information

Supplementary Information.

## Data Availability

The datasets used and/or analyzed during the current study available from the corresponding author on reasonable request.

## References

[1] Li K, Feng M, Biswas A, Su H, Niu Y, Cao J (2020). Driving factors and future prediction of land use and cover change based on satellite remote sensing data by the LCM model: A case study from Gansu province, China. Sensors.

[2] Mitsch WJ, Gosselink JG (2015). Wetlands.

[3] Eskandari Damaneh H, Khosravi H, Habashi K, Eskandari Damaneh H, Tiefenbacher JP (2022). The impact of land use and land cover changes on soil erosion in western Iran. Nat. Hazards.

[4] Ghoochani OM, Eskandari Damaneh H, Eskandari Damaneh H, Ghanian M, Cotton M (2023). Why do farmers over-extract groundwater resources? Assessing (un) sustainable behaviors using an Integrated Agent-Centered framework. Environments.

[5] Makrouni S, Sabzghabaei GR, Yousefi Khanghah S, Soltanian S (2016). Detection of land use changes in Hoor Al Azim wetland using remote sensing and geographic information system techniques. J. RS GIS Nat. Resour..

[6] Cui L, Li G, Liao H, Ouyang N, Zhang Y (2015). Integrated approach based on a regional habitat succession model to assess wetland landscape ecological degradation. Wetlands.

[7] Hill, J., Jarmer, T., Udelhoven, T., & Stellmes, M. Remote Sensing and Geomatics Applications for Desertification and Land Degradation Monitoring and Assessment. *Geometrics for land and water management: Achievements and challenges in the Euromed context (June 2014)* 15–22 (2006).

[8] Hu T, Liu J, Zheng G, Zhang D, Huang K (2020). Evaluation of historical and future wetland degradation using remote sensing imagery and land use modeling. Land Degrad. Dev..

[9] Abd El-Kawy OR, Rød JK, Ismail HA, Suliman AS (2011). Land use and land cover change detection in the western Nile delta of Egypt using remote sensing data. Appl. Geogr..

[10] van de Nick, G, Paul LG, Park SJ (2005). Optimal spatial scale for land use change modelling: A case study in a savanna landscape in Northern Ghana. J. Korean Geogr. Soc..

[11] Oñate-Valdivieso F, Sendra JB (2010). Application of GIS and remote sensing techniques in generation of land use scenarios for hydrological modeling. J. Hydrol..

[12] Schulz JJ, Cayuela L, Echeverria C, Salas J, Benayas JMR (2010). Monitoring land cover change of the dryland forest landscape of Central Chile (1975–2008). Appl. Geogr..

[13] Khoi DD, Murayama Y (2010). Forecasting areas vulnerable to forest conversion in the Tam Dao National Park Region. Vietnam. Remote Sens..

[14] Václavík T, Rogan J (2009). Identifying trends in land use/land cover changes in the context of post-socialisttransformation in Central Europe: A case study of the greater Olomouc region, Czech Republic. GISci & Remote Sens..

[15] Gemitzi A (2021). Predicting land cover changes using a CA Markov model under different shared socioeconomic pathways in Greece. GIScience Remote Sens..

[16] Koko AF, Yue W, Abubakar GA, Hamed R, Alabsi AAN (2020). Monitoring and predicting spatio-temporal land use/land cover changes in Zaria City, Nigeria, through an integrated cellular automata and markov chain model (CA-Markov). Sustainability (Switzerland).

[17] Ansari A, Golabi MH (2019). Prediction of spatial land use changes based on LCM in a GIS environment for Desert Wetlands–A case study: Meighan Wetland, Iran. Int. Soil Water Conserv. Res..

[18] Gupta R, Sharma LK (2020). Efficacy of Spatial Land Change Modeler as a forecasting indicator for anthropogenic change dynamics over five decades: A case study of Shoolpaneshwar Wildlife Sanctuary, Gujarat, India. Ecol. Indic..

[19] Joorabian Shooshtari S, Aazami J (2023). Prediction of the dynamics of land use land cover using a hybrid spatiotemporal model in Iran. Environ. Monit. Assess..

[20] Khoshnood Motlagh S, Sadoddin A, Haghnegahdar A, Razavi S, Salmanmahiny A, Ghorbani K (2021). Analysis and prediction of land cover changes using the land change modeler (LCM) in a semiarid river basin, Iran. Land Degrad. Dev..

[21] Leta MK, Demissie TA, Tränckner J (2021). Modeling and prediction of land use land cover change dynamics based on land change modeler (Lcm) in Nashe watershed, upper blue Nile basin, Ethiopia. Sustainability.

[22] Roushangar K, Alami MT, Golmohammadi H (2023). Modeling the effects of land use/land cover changes on water requirements of Urmia Lake basin using CA-Markov and NETWAT models. Model. Earth Syst. Environ..

[23] Thapa RB, Murayama Y (2011). Urban growth modeling of Kathmandu metropolitan region, Nepal. Comput. Environ. Urban Syst..

[24] Megahed Y, Cabral P, Silva J, Caetano M (2015). Land cover mapping analysis and urban growth modelling using remote sensing techniques in Greater Cairo Region—Egypt. ISPRS Int. J. Geo-Inf..

[25] Wang Q, Wang H, Chang R, Zeng H, Bai X (2022). Dynamic simulation patterns and spatiotemporal analysis of land-use/land-cover changes in the Wuhan metropolitan area China. Ecol. Model..

[26] Arora A, Pandey M, Mishra VN, Kumar R, Rai PK, Costache R (2021). Comparative evaluation of geospatial scenario-based land change simulation models using landscape metrics. Ecol. Ind..

[27] Kourosh Niya A, Huang J, Kazemzadeh-Zow A, Karimi H, Keshtkar H, Naimi B (2020). Comparison of three hybrid models to simulate land use changes: A case study in Qeshm Island, Iran. Environ. Monitor. Assess..

[28] Camacho Olmedo MT, Pontius RG, Paegelow M, Mas J-F (2015). Comparison of simulation models in terms of quantity and allocation of land change. Environ. Model. Softw..

[29] Kosmas C (2014). Evaluation and selection of indicators for land degradation and desertification monitoring: methodological approach. Environ. Manag..

[30] Shen G, Yang X, Jin Y, Xu B, Zhou Q (2019). Remote sensing and evaluation of the wetland ecological degradation process of the Zoige Plateau Wetland in China. Ecol. Indic..

[31] Das A, Basu T (2020). Assessment of peri-urban wetland ecological degradation through importance-performance analysis (IPA): A study on Chatra Wetland, India. Ecol. Indic..

[32] Azareh A, Sardooi ER, Gholami H, Mosavi A, Shahdadi A, Barkhori S (2021). Detection and prediction of lake degradation using landscape metrics and remote sensing dataset. Environ. Sci. Pollut. Res..

[33] Peng K, Jiang W, Wang X, Hou P, Wu Z, Cui T (2023). Evaluation of future wetland changes under optimal scenarios and land degradation neutrality analysis in the Guangdong-Hong Kong-Macao Greater Bay Area. Sci. Total Environ..

[34] Pournabi N, Janatrostami S, Ashrafzadeh A, Mohammadi K (2021). Resolution of Internal conflicts for conservation of the Hour Al-Azim wetland using AHP-SWOT and game theory approach. Land Use Policy.

[35] Ahmadzadeh Hadith (2016). Evapotranspiration Estimation Based on the SEBAL Model in the Hoor Al-Azeem Wetland.

[36] Zibanchi, M., Naseri, H., Ali, A., Nematullah, J. H. & Bina, M. (2018). Explanation of a quantitative and qualitative balance model in determining the status of wetlands (Horul Azim wetland). In *The Third Conference and Specialized Exhibition of Environmental Engineering *8. https://civilica.com/doc/68651.

[37] Salmabai, H. & Saeedi, M. Areal fluctuations monitoring of Al-Azim/Al-Havizeh wetland during the 1986–2017 period, using time-series Landsat data. In *The 2 nd International Conference on Strategic Ideas for Architecture Urbanism, Geography, and the Environment, Mashhad, Iran* (2018).

[38] Mayahi J, Eskandari dameneh H, Zarasvandi A (2021). Assessments land cover change effects on soil erosion trend in Hoor al-Azim wetland, Southwestern of Iran. J. Nat. Environ. Hazards.

[39] Nadaf, S. *The Use of Satellite Images to Assess the Risks of Changes in Land Use in Wetland Landscape HUROL’AZIM*. University of Tabriz (2016).

[40] Dezhkam S, Jabbarian Amiri B, Darvishsefat AA, Sakieh Y (2017). Performance evaluation of land change simulation models using landscape metrics. Geocarto Int..

[41] Abdelkareem OEA, Elamin HMA, Eltahir MES, Adam HE, Elhaja ME, Rahamtalla AM (2018). Accuracy assessment of land use land cover in Umabdalla natural reserved forest, South Kordofan, Sudan. Int. J. Agric. Environ. Sci..

[42] Munsi M, Areendran G, Joshi PK (2012). Modeling spatio-temporal change patterns of forest cover: A case study from the Himalayan foothills (India). Reg. Environ. Change.

[43] Joorabian Shooshtari S, Shayesteh K, Gholamalifard M, Azari M, López-Moreno JI (2021). Responses of surface water quality to future land cover and climate changes in the Neka River basin, Northern Iran. Environ. Monitor. Assess..

[44] Tiné M, Perez L, Molowny-Horas R, Darveau M (2019). Hybrid spatiotemporal simulation of future changes in open wetlands: A study of the Abitibi-Témiscamingue region, Québec, Canada. Int. J. Appl. Earth Obs. Geoinf..

[45] Rahman MT, Aldosary AS, Mortoja MG (2017). Modeling future land cover changes and their effects on the land surface temperatures in the Saudi Arabian eastern coastal city of Dammam. Land.

[46] Heidarlou HB, Shafiei AB, Erfanian M, Tayyebi A, Alijanpour A (2019). Effects of preservation policy on land use changes in Iranian Northern Zagros forests. Land Use Policy.

[47] Eastman JR (2015). TerrSet: Geospatial Monitoring and Modeling Software.

[48] Sangermano F, Toledano J, Eastman JR (2012). Land cover change in the Bolivian Amazon and its implications for REDD+ and endemic biodiversity. Landsc. Ecol..

[49] Eastman JR (2012). IDRISI Selva Tutorial. Idrisi Prod. Clark Labs-Clark Univ..

[50] Pérez-Vega A, Mas J-F, Ligmann-Zielinska A (2012). Comparing two approaches to land use/cover change modeling and their implications for the assessment of biodiversity loss in a deciduous tropical forest. Environ. Model. Softw..

[51] Islam K, Rahman MF, Jashimuddin M (2018). Modeling land use change using cellular automata and artificial neural network: The case of Chunati Wildlife Sanctuary, Bangladesh. Ecol. Indic..

[52] Sang L, Zhang C, Yang J, Zhu D, Yun W (2011). Simulation of land use spatial pattern of towns and villages based on CA–Markov model. Math. Comput. Model..

[53] Yirsaw E, Wu W, Shi X, Temesgen H, Bekele B (2017). Land use/land cover change modeling and the prediction of subsequent changes in ecosystem service values in a coastal area of China, the Su-Xi-Chang region. Sustainability.

[54] Houet, T. & Hubert-moy, L. Modelling and projecting land-use and land-cover changes with a cellular automaton in considering landscape trajectories: An improvement for simulation of plausible future states. In *EASeL eProceedings* 63–76 (2006).

[55] Naboureh A, Rezaei Moghaddam MH, Feizizadeh B, Blaschke T (2017). An integrated object-based image analysis and CA-Markov model approach for modeling land use/land cover trends in the Sarab plain. Arab. J. Geosci..

[56] Haji Moradi, A. & Najib, H. *Examining the Performance of the “Provisions of the Sixth Development Plan Regarding the Protection, Restoration, Management, and Proper Exploitation of the Country’s Wetlands” (Part “B” of Article 38 (Sixth Plan)).* (No. 18291) (2022).

[57] Al-Lami A (2012). Action Plan for Implementing the Programme of Work on Protected Areas of the Convention on Biological Diversity, Iraq.

[58] Birch CPD, Oom SP, Beecham JA (2007). Rectangular and hexagonal grids used for observation, experiment and simulation in ecology. Ecol. Model..

[59] Irankhahi M, Jozi A (2016). The lattice hexagon approach applied in landscape degradation assessment (Case study: Shemiranat County). Town Country Plan.

[60] Sadeghi Benis M (2015). Using landscape metrics in rehabilitation of urban ecological network. Mon. Sci. J. Bagh-e Nazar.

[61] Tian Y, Jim CY, Tao Y, Shi T (2011). Landscape ecological assessment of green space fragmentation in Hong Kong. Urban For. Urban Green..

[62] Mohammadi A, Fatemizadeh F (2021). Quantifying landscape degradation following construction of a highway using landscape metrics in southern Iran. Front. ecol. evol..

[63] McGarigal, K., & Marks, B. J. Spatial pattern analysis program for quantifying landscape structure. *Gen. Tech. Rep. PNW-GTR-351. US Department of Agriculture, Forest Service, Pacific Northwest Research Station* (1995).

[64] Adger WN (2006). Vulnerability. Glob. Environ. Chang..

[65] Yu W, Zang S, Wu C, Liu W, Na X (2011). Analyzing and modeling land use land cover change (LUCC) in the Daqing City, China. Appl. Geogr..

[66] Zhu P, Gong P (2014). Suitability mapping of global wetland areas and validation with remotely sensed data. Sci. China Earth Sci..

[67] Jamei, M., Hammadi, K., SeyyedMohsen, H. & AlaeiRozbahani, R. Investigating the status of water reserves of Horul Azim lagoon using remote sensing techniques. In *Geomatics Conference 86*. https://civilica.com/doc. (2016).

[68] Ghorbanian Gabraail KP (2018). Study of Ahvaz Dust Textures With of X-Ray analysing method and the relationship between storms exacerbated by destruction of hoorolazim wetlands. Wetland Ecobiol..

[69] Wang R, Murayama Y (2017). Change of land use/cover in Tianjin City based on the Markov and cellular automata models. ISPRS Int. J. Geo-Inf..

[70] Zabihi M, Moradi H, Gholamalifard M, Khaledi Darvishan A, Fürst C (2020). Landscape management through change processes monitoring in Iran. Sustainability.

[71] Shoyama K (2021). Assessment of land-use scenarios at a national scale using intensity analysis and figure of merit components. Land..

[72] Basu T, Das A, Pham QB, Al-Ansari N, Linh NTT, Lagerwall G (2021). Development of an integrated peri-urban wetland degradation assessment approach for the Chatra Wetland in eastern India. Sci. Rep..

[73] Savari M, Damaneh HE, Damaneh HE, Cotton M (2023). Integrating the norm activation model and theory of planned behaviour to investigate farmer pro-environmental behavioural intention. Sci. Rep..

[74] Savari M, Damaneh HE, Damaneh HE (2023). Effective factors to increase rural households' resilience under drought conditions in Iran. Int. J. Disast. Risk Reduct..

[75] Voight C, Hernandez-Aguilar K, Garcia C, Gutierrez S (2019). Predictive modeling of future forest cover change patterns in southern Belize. Remote Sens..

