# Supplementary material for: Estimating the severity of landscape degradation in future management scenarios based on modeling the dynamics of Hoor Al-Azim International Wetland in Iran-Iraq border
Source: Sci Rep. 2024 May 24;14:11877. doi: 10.1038/s41598-024-62649-0 (PMC11126657; doi:10.1038/s41598-024-62649-0)
Supplement: Supplementary file 1 — Supplementary Information. [file 41598_2024_62649_MOESM1_ESM.pdf]

Supplementary Table 1: Driver variables used in the model

Variable	Scale	Source	Year
DEM	1:120000	ASTER digital elevation model	2019
Slope	1:120000	DEM	2019
Proximity to main roads	1:25,000	topographic maps	1985, 2000
Proximity to rivers	1:25,000	topographic maps	1985, 2000
Proximity to sparse vegetation	1:120000	land cover map	1985, 2000
Proximity to dense vegetation	1:120000	land cover map	1985, 2000
Proximity to humid lands	1:120000	land cover map	1985, 2000
Proximity to water bodies	1:120000	land cover map	1985, 2000
Proximity to salt lands	1:120000	land cover map	1985, 2000
evidence likelihood to change map	1:120000	land cover map	1985, 2000
Annual average precipitation	1 Km	Global climate and weather data	1970-2000
Annual average temperature	1 Km	Global climate and weather data	1970-2000

Supplementary Table 2: Error matrix of land cover classification

**Confusion matrix: 1985**						
LULC types	Sparse vegetation	Water bodies	Humid land	Dense vegetation	Salt	Error Commission
Sparse vegetation	47	2	3	3	3	**0.189655**
Water bodies	1	49	1	3	1	**0.109091**
Humid land	2	2	43	1	2	**0.140000**
Dense vegetation	1	0	0	44	0	**0.022222**
Salt	1	1	3	0	45	**0.100000**
Error Omission	**0.096154**	**0.092593**	**0.140000**	**0.137255**	**0.117647**	**0.116279**
**Confusion matrix: 2000**						
LULC types	Sparse vegetation	Water bodies	Humid land	Dense vegetation	Salt	Error Commission
Sparse vegetation	47	2	1	2	0	**0.096154**
Water bodies	1	45	0	2	0	**0.062500**
Humid land	0	0	48	0	3	**0.058824**
Dense vegetation	1	1	2	47	0	**0.078431**
Salt	1	0	1	1	48	**0.058824**
Error Omission	**0.060000**	**0.062500**	**0.076923**	**0.096154**	**0.058824**	**0.071146**
**Confusion matrix: 2015**						
LULC types	Sparse vegetation	Water bodies	Humid land	Dense vegetation	Salt	Error Commission
Sparse vegetation	52	3	4	2	1	**0.161290**
Water bodies	0	53	1	0	0	**0.018519**
Humid land	2	1	44	0	0	**0.063830**
Dense vegetation	4	1	0	44	2	**0.137255**
Salt	0	0	2	0	43	**0.044444**
Error Omission	**0.103448**	**0.086207**	**0.137255**	**0.043478**	**0.065217**	**0.088803**

Supplementary Table 3: The results of evaluating the accuracy of transition potential modeling with artificial neural network

Sub-model	Accuracy rate (%)	Pierce Skill
Water to Sparse vegetation	90.23	0.8047
Water to Dense vegetation	83.17	0.6635
Water to Humid land	89.51	0.7902
Water to Salt	98.04	0.9608
Humid land to Sparse vegetation	83.35	0.6669
Dense vegetation to Sparse vegetation	82.22	0.6444
Dense vegetation to Humid land	87.27	0.7454

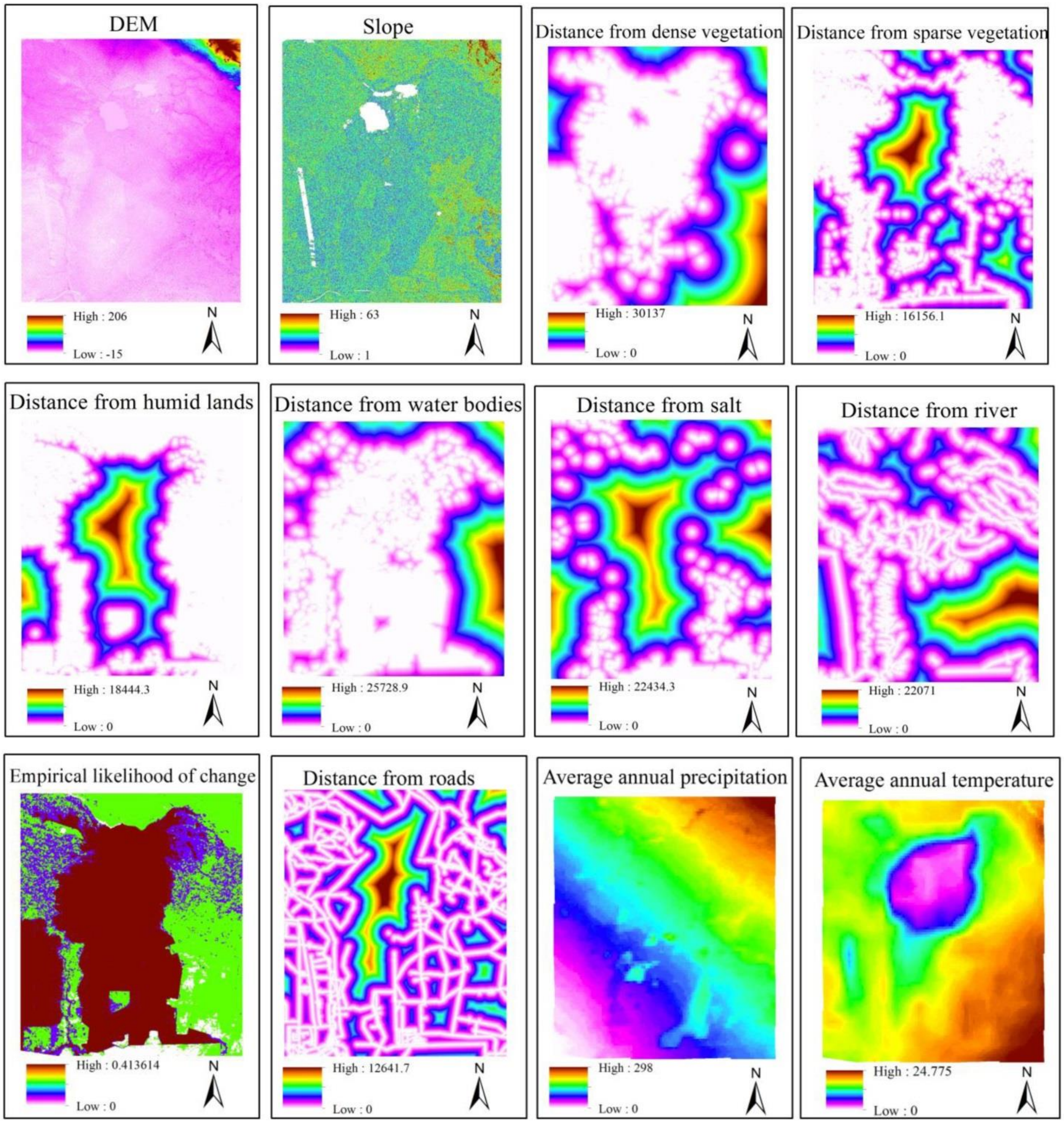

Supplementary Figure 1: Variables used in transition potential modeling

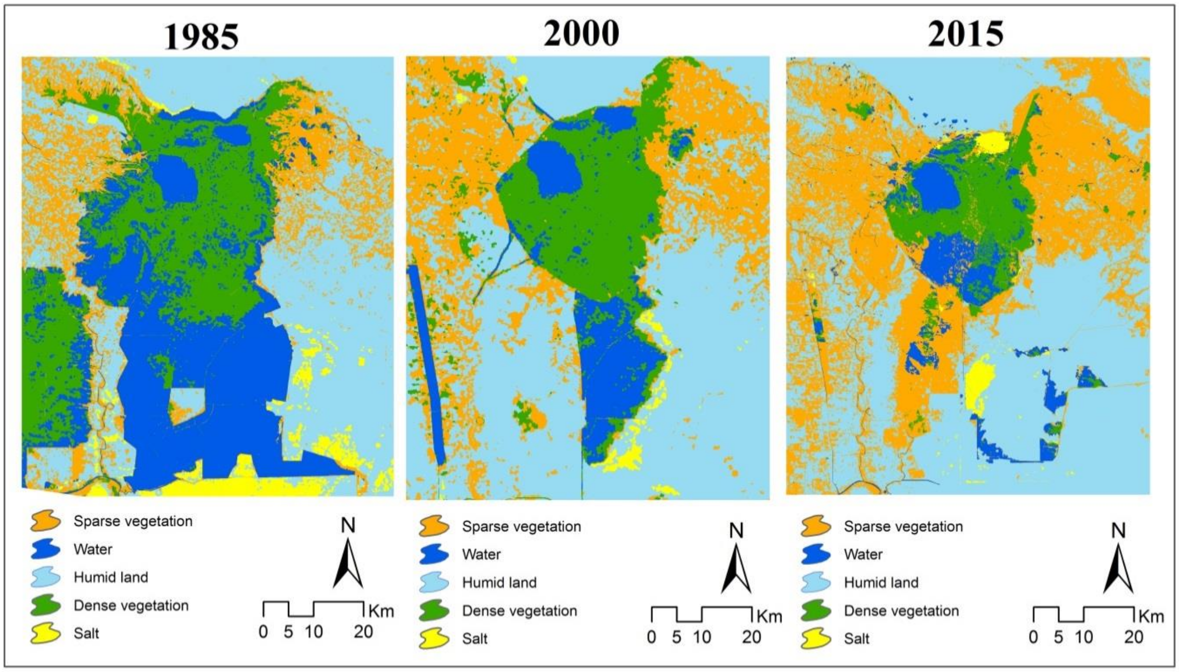

Supplementary Figure 2: Land cover maps of Hoor Al-Azim International Wetland in 1985, 2000, and 2015

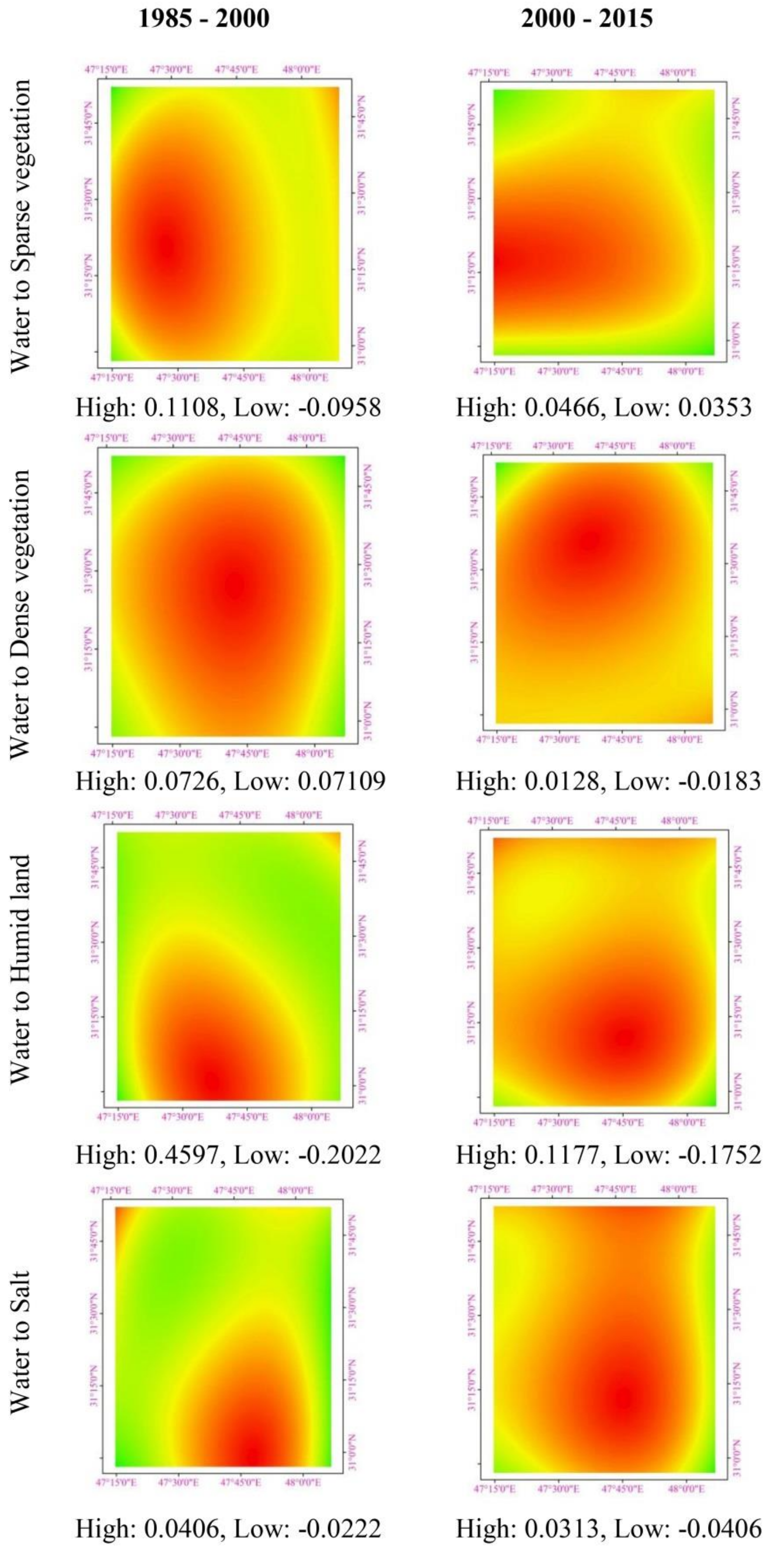

Supplementary Figure 3: Spatial trend of change maps from water to other land cover (red areas show higher numbers, and green areas show lower numbers).

Analysis of changes in land covers

Land cover changes during 1985–2000: The water bodies were 25.1% of the entire area in 1985 and decreased by 7.1% in 2000, for an annual rate of 8.3%. The rate of reduction in the water was 148,139 ha during 1985–2000 (Supplementary Table 4). Sparse vegetation increased from 14.8% of the study area to 24.3%, with an annual rate of 3.3%. Humid lands also increased from 297,122 ha in 1985 to 453,931 ha in 2000. The annual rate of increase in this class was 2.82%. From 1985 to 2000, dense vegetation, and salt lands demonstrated a decrease with an annual rate of 2.9% and 9.7%, respectively. 28,729, 104,551, 8,839, and 5,633 ha of water bodies were changed to sparse vegetation, humid lands, dense vegetation, and salt lands, respectively. The amount of net change from dense vegetation to humid lands, and sparse vegetation is 42,563 and 24806 ha, respectively. 22,512 ha of humid lands were changed to sparse vegetation. The gain and loss in water bodies were 15,735 ha and – 163,487 ha, respectively.

Supplementary Table 4: The area and changes of different land use in the studied years in the Hoor Al-Azim Wetland

Year	1985		2000		2015		Area changed	
Land cover class	ha	%	ha	%	ha	%	1985-2000	2000-2015
Sparse vegetation	122644.08	14.86	202393.89	24.38	293903.64	35.56	+79749.8	+91509.7
Water	207511.02	25.15	59371.38	7.15	50264.28	6.08	-148139.6	-9107.1
Humid land	297122.04	36.01	453931.56	54.70	409075.29	49.50	+156809.5	-44856.2
Dense vegetation	164614.32	19.95	106439.22	12.82	63508.95	7.68	-58175.1	-42930.2
Salt	33181.92	4.02	7703.46	0.92	9628.56	1.16	-25478.4	+1925.1

Land cover changes during 2000–2015: Sparse vegetation increased during this period at an annual rate of 2.5% and 91,509 ha. From 2000 to 2015, the net change from dense vegetation, water, and salt lands to sparse vegetation was 18,034, 9985, and 62,946 ha, respectively. Water bodies decreased by 9107 ha at an annual rate of 1.1%. The net change from water to humid and salt lands was 9741 and 7449 ha, respectively. A decrease in dense vegetation of 42,930 ha (from 12.8% of the total area in 2000 to 7.6% in 2015) was shown at an annual rate of 3.4%. The net change from dense vegetation to water, humid lands, and salt lands was 18172, 6202, and 87 ha, respectively. During this period, salt lands increased from 7703 ha in 2000 to 9628 ha in 2015 at an annual rate of 1.5%. According to the spatial trend map (Supplementary Fig. 3), the most changes from water to salt lands occurred in the region's south during 1985–2000 and in the center and south of the studied region during 2000–2015. Pixels with the highest change from water to sparse vegetation were also observed in the western part of the region.
